# Atom probe tomography data analysis procedure for precipitate and cluster identification in a Ti-Mo steel

**DOI:** 10.1016/j.dib.2018.03.094

**Published:** 2018-03-27

**Authors:** S. Dhara, R.K.W. Marceau, K. Wood, T. Dorin, I.B. Timokhina, P.D. Hodgson

**Affiliations:** a Deakin University, Institute for Frontier Materials, Geelong, VIC 3216, Australia; b Australian Nuclear Science and Technology Organisation (ANSTO), Kirrawee, New South Wales, Australia

## Abstract

An atom probe tomography data analysis procedure for identification of particles in a Ti-Mo steel is presented. This procedure has been used to characterise both carbide precipitates (larger particles) and solute clusters (smaller particles), as reported in an accompanying Mater. Sci. Eng. A paper [1]. Particles were identified using the maximum separation method (cluster-finding algorithm) after resolving peak overlaps at several locations in the mass spectrum. The cluster-finding algorithm was applied to the data in a two-stage process to properly identify particles having a bimodal size distribution. Furthermore, possible misidentification of matrix atoms (mainly Fe) due to the local magnification effect (from the difference in field evaporation potential between the matrix and precipitates) has been resolved using an atomic density approach, comparing that measured experimentally using APT to the theoretical density of both the matrix and particles.

**Specifications table**

**Table:** 

Subject area	*Materials science*
More specific subject area	*Particle discrimination using atom probe tomography*
Type of data	*Tables, images, graphs*
How data was acquired	*Atom probe tomography (APT)*
Data format	*Post-processed (reconstructed) APT data*
Experimental factors	*Samples for APT were electropolished.*
Experimental features	*APT experiments were performed using a LEAP 4000 HR instrument (CAMECA Instruments Inc.) in voltage pulsing mode under ultrahigh vacuum at a set-point temperature of 60 K, using a pulse fraction of 20%, pulse repetition rate of 200 kHz and detection rate of 0.005 atoms per pulse. Reconstruction, visualisation and analysis of the APT data were performed using the IVAS 3.6.12 software.*
Data source location	*Deakin University, Institute for Frontier Materials, Geelong, VIC 3216, Australia*
Data accessibility	*Data included in the article*

**Value of the data**

• Accurate determination of chemical composition is important for subsequent APT data analysis (e.g. cluster-finding).
• The cluster-finding analysis procedure is readily available to APT users with access to the most common commercial software (IVAS) for APT data analysis.
• The procedure follows a multi-stage approach so it can be applied to any system containing particles of multiple size ranges.
• Identification of matrix atoms within precipitates can be complex due to atom probe artefacts. The atomic density approach developed here provides new insight towards the correction of chemical composition of precipitate particles whose detection using APT is influenced by the local magnification effect caused by the difference in field evaporation potential between the matrix and precipitates.

## 1 Data, materials and methods

This article presents the APT data analysis procedure used in [1]. The data analysis was performed to identify both precipitate carbides and clustered solute atoms in the atom probe data collected from a 0.04C-1.5Mn-0.1Ti-0.2Mo wt% (0.19C-1.5Mn-0.1Ti-0.1Mo at%) steel that had undergone various thermomechanical processing treatments. Details regarding the steel and processing conditions can be found in [1]. Experimental APT data collection parameters are given in the Specifications Table above.

## 2 Experimental data analysis

Analysis of the (reconstructed) experimental APT data has been carried out in various steps. Firstly, the chemical composition of each dataset has been determined as accurately as possible to ensure opportunity for accurate analyses that follow (e.g. cluster-finding analysis). Here, we deal with the issue of peak overlap in the APT mass spectrum. The next step is cluster-finding analysis which involves determination of the cluster-finding algorithm input parameters in an optimised way. This second step is a two-stage approach, designed to properly identify particles that exhibit a bimodal size distribution, i.e. separate identification of firstly the carbide precipitates, and secondly the solute clusters within the matrix solid solution. With respect to carbide composition determination, post-identification by cluster-finding analysis, we present an approach to account for the possible misidentification of matrix atoms (mainly Fe) due to the local magnification effect that arises from the difference in field evaporation potential between the matrix and precipitates. This approach is based on measurement of the atomic densities of the matrix and carbide particles in the APT data, as compared to their respective theoretical densities, and allows for better assessment of the local chemical composition of the particles.

### 2.1 Estimation of chemical composition

**Fig. 1:**
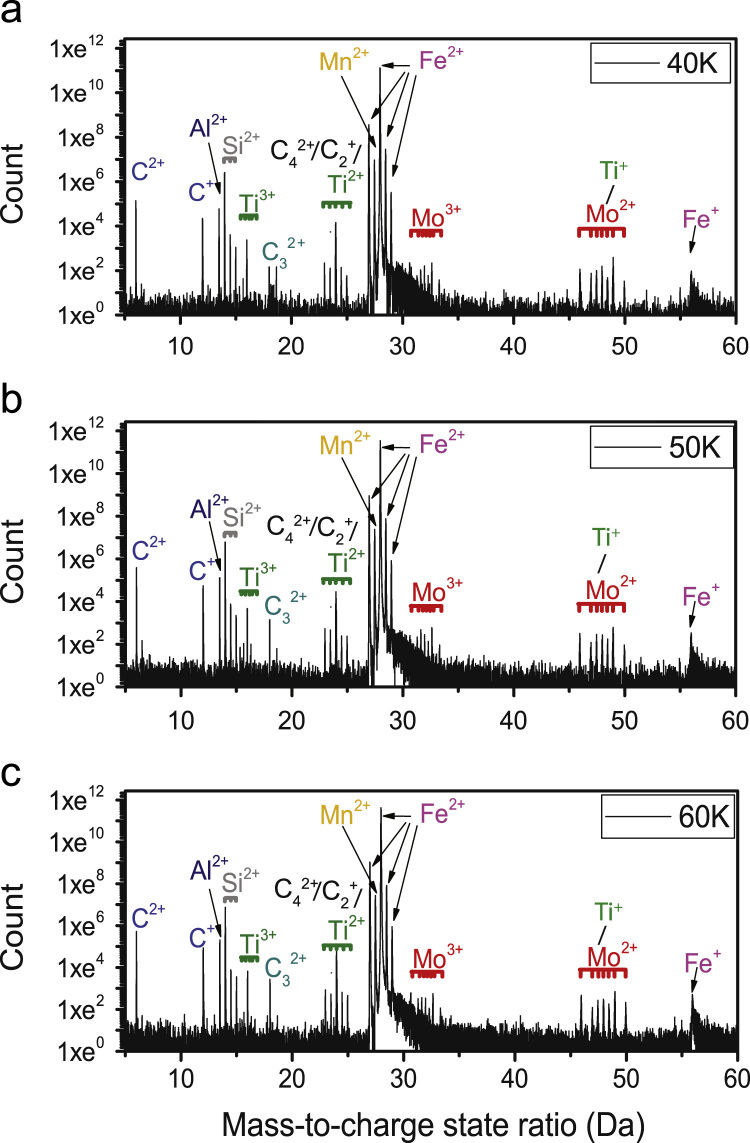
Mass spectrum of the sample 1 h – S1 obtained from experiments performed at (a) 40 K, (b) 50 K and (c) 60 K.

The objective of the atom probe experiments in the present work is to properly identify precipitates and clusters present in the steel subjected to various thermomechanical processing conditions. The initial step towards the data analysis procedure was therefore determination of chemical composition as accurately as possible, which is achieved by identifying the mass spectrum peaks according to the various charge states and abundance of elemental isotopes. A typical mass spectrum of the steel is shown in Fig. 1a.

As indicated in Fig. 1a, peak overlap occurs at 24 Da (Ti^2+^, C^2+^ and C_4_^2+^) and between 46 - 50 Da for isotopes of Ti^+^ and Mo^2+^. The latter conflict has been resolved using the peak decomposition tool in the IVAS software, where the assignment of peaks is decided by comparing the decomposed abundance with the expected abundance of the elemental isotopes in question. The results of the decomposed abundance have been presented in Table 1. Clearly, the decomposed abundance of Ti^+^ matches with the expected abundance at the 46 and 48 Da peaks, but the values are either lower or higher compared to the expected abundance for the other three peaks. In contrast, the decomposed abundance of all the Mo^2+^ peaks are in good agreement with the expected abundance for all the isotopes, and so the peaks in the 46 – 50 Da range have been designated to Mo^2+^ since it is the largest contributor in that range. It is essential to designate every peak in the mass spectrum with a single ion for further cluster finding analyses.

**Table 1:** Peak decomposition for Ti^+^ and Mo^2+^ ions in the 46–50 Da range.

**Ion**	**Range**	**Decomposed abundance**	**Expected abundance**	**Estimated error**
**Mo^2+^**	45.85 – 46.03	0.1474	0.1477	0.0037
**Ti^2+^**		0.0848	0.0825	0.0883
				
**Mo^2+^**	46.91 – 47.01	0.0902	0.0923	0.0041
**Ti^2+^**		5.28 × 10^–11^	0.0744	0.0539
				
**Mo^2+^**	47.88 – 48.03	0.1662	0.1668	7.25 × 10^-4^
**Ti^2+^**		0.7031	0.7372	0.1253
				
**Mo^2+^**	48.87 – 49.03	0.2428	0.2419	0.0042
**Ti^2+^**		0.1133	0.0541	0.1046
				
**Mo^2+^**	49.87 – 50.01	0.0976	0.0967	0.0022
**Ti^2+^**		0.0987	0.0518	0.0914

The expected and decomposed abundance of the various overlapping ions in the 24 – 25 Da range have been shown in Table 2. While it is clear that Ti^2+^ is the only element having a decomposed abundance closely matching to that expected in all three instances, contribution from the other two molecular ions (C_2_^+^ and C_4_^2+^) is also crucial for the present case. This is because the ion selection at 24 Da will decide the total amount of Ti and C in the sample, which are the primary contributors to the chemical composition of the precipitates in the present case. Therefore, we also need to carefully consider the experimental run parameters used to gather the data to study their effect on the mass spectrum before deciding on the 24 Da peak.

**Table 2:** Peak decomposition for Ti^2+^, C_2_^+^ and C_4_^2+^ ions in the 24 – 25 Da range.

**Ion**	**Range (Da)**	**Decomposed abundance**	**Expected abundance**	**Estimated error**
**Ti^2+^**	23.93 – 24.07	0.7364	0.7372	0.0009
**C_2_^+^**		1.04 × 10^–17^	0.9779	0.4596
**C_4_^2+^**		0.8972	0.9564	0.3215
				
**Ti^2+^**	24.45 – 24.52	0.0549	0.0541	0.002
**C_2_^+^**		No peak		
**C_4_^2+^**		0.0526	0.0429	0.0377
				
**Ti^2+^**	24.95 – 25.03	0.0535	0.0518	0.0016
**C_2_^+^**		1	0.0219	0.4582
**C_4_^2+^**		0.0233	7.20 × 10^-4^	0.1741

Takahashi et al. demonstrated that the apparent carbon concentration does not change by varying the pulse fraction [2]. However, the unwindowed signal-to-noise ratio (background noise) has been observed to increase at lower values (such as 15% pulse fraction) due to increase in field evaporation of ions during DC voltage operation [2]. The effect of pulse fraction has been reported to be insignificant on the desorption images [3]. However, Yao et al. did observe a strong dependence of temperature on the chemical composition, signal-to-noise ratio and desorption images for data produced from microalloyed steels [3]. They concluded that the optimum experimental temperature for microalloyed steels should be around 20 K, and they also proposed that lower experimental temperature provides better possibility for identification of molecular ions that tend to occur on multiple hit events. Despite this, increased chances of specimen rupture at such lower temperature also needed to be considered. In the current experimental work, the pulse fraction was kept at 20% to minimise background noise. To decide on temperature, experiments were performed at 40 K, 50 K and 60 K on a single specimen, with approximately 5 million ions collected for each condition. The reconstruction parameters were kept constant for comparison purpose. Primarily, the effect of temperature on the appearance of molecular ion peaks and the chemical composition has been considered.

**Fig. 2:**
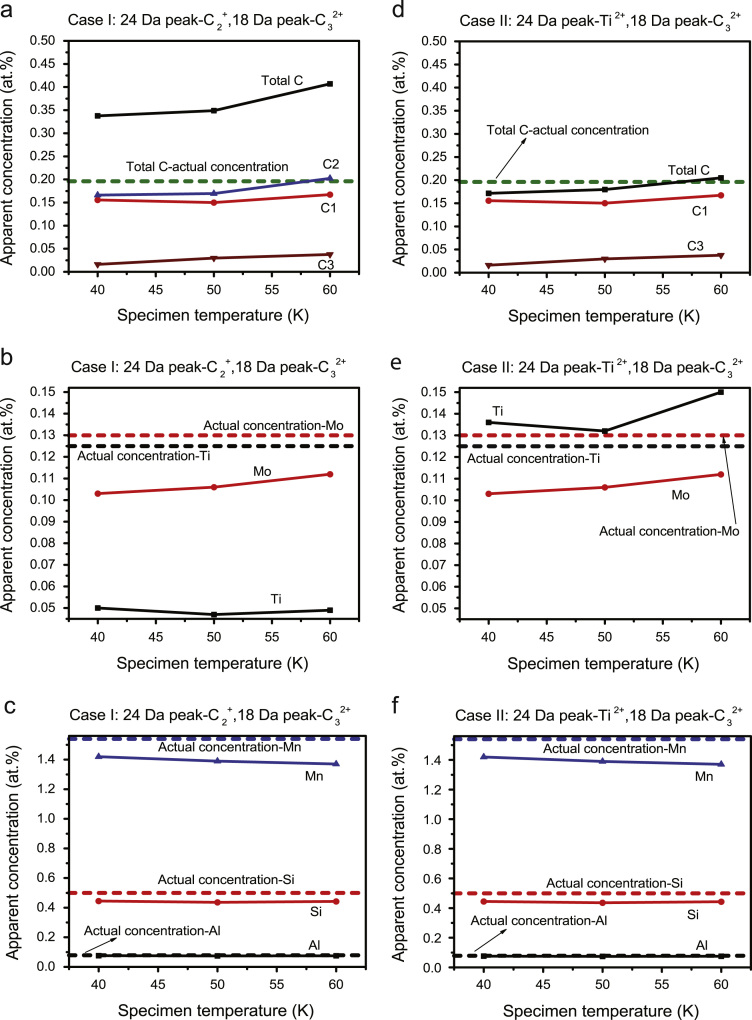
Effect of temperature and peak assignment on the chemical composition (a-c) 24 Da peak assigned to C_2_^+^ (d-f) 24 Da peak assigned to Ti^2+^.

The mass spectra obtained at different temperatures are shown in Fig. 1a-c. The background noise is visibly improved at 40 K, but there is no significant difference between the mass spectra obtained from 50 K and 60 K conditions. Chemical composition has also been determined for these different conditions. As mentioned earlier, the 24 Da peak has an overlap between Ti^2+^ and C_2_^+^, and possibly also C_4_^2+^, however the chemical composition at different temperatures has only been evaluated by assigning this peak as either C_2_^+^ (Case I) or Ti^2+^ (Case II), and the C_4_^2+^ ion is not considered in further analysis. The reason for this will be discussed at the end of the next paragraph. Fig. 2a-c presents the atomic concentration of each element as a function of temperature for Case I, and similarly for Case II in Fig. 2d-f. The dotted lines in the plots represent the bulk composition measured by spark emission spectroscopy.

Fig. 2a and d shows the effect of experimental temperature on the C composition for Case I and II, respectively. The solid black line represents the total C composition, the coloured solid lines show the decomposed amount of different forms of detected molecular C ions. It is evident from these plots that the amount of total C is overestimated in Case I irrespective of experimental temperature. If we consider the decomposed amount of the molecular C ions, it is clear that the C_1_ and C_3_ ion contents are similar in both cases. Additionally, the estimated amount of total C agrees with the actual C composition (dotted line). Therefore, introducing C_2_ in the mass spectrum is the reason for overestimation of total C in Case I. The plots in Fig. 2b and 2e support the same fact, where the amount of Ti is underestimated in Case I. The concentration of the other elements (Mn, Si and Al) remained constant in Case I and II (Fig. 2c and f). Therefore, it is easily understood that the total C composition would be significantly overestimated if the 24 Da peak were assigned to C_4_^2+^, and as such it has not been considered further in this analysis. In summary, the peak at 24 Da has been assigned to Ti^2+^.

### 2.2 Cluster-finding analysis for precipitates

The cluster-finding analysis has been performed on the APT data using the cluster analysis tool in IVAS 3.6.12 software [4]. The analysis is based on the maximum separation algorithm [5], which uses two user-defined parameters, *d*_max_ (maximum distance between atoms in a cluster) and *N*_min_ (minimum number of atoms in a cluster). The *d*_max_ parameter has been determined by using nearest neighbour (NN) distance frequency distribution as a heuristic tool. The order (*K*) of the NN distribution determines the size range of the cluster to be found. Larger values of *K* ignore the local density fluctuations compared to smaller *K*, and thus, identify only the larger features [6]. Therefore, three major parameters needed to be identified in the present analysis; *d*_max_, *N*_min_ and *K*. The NN distribution obtained from the experimental data was compared to that from random data, following Stephenson et al. [6]. The random data has been generated by swapping the chemical identities of each atom but keeping the atomic positions the same, a process known as random labelling (RL) [6].

**Fig. 3:**
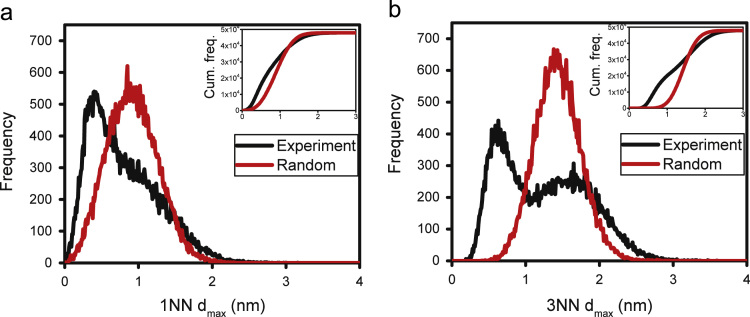
KNN frequency distributions for Ti, Mo and C atoms; (a) 1NN and (b) 3NN distance. Inset shows corresponding cumulative frequency distribution.

**Fig. 4:**
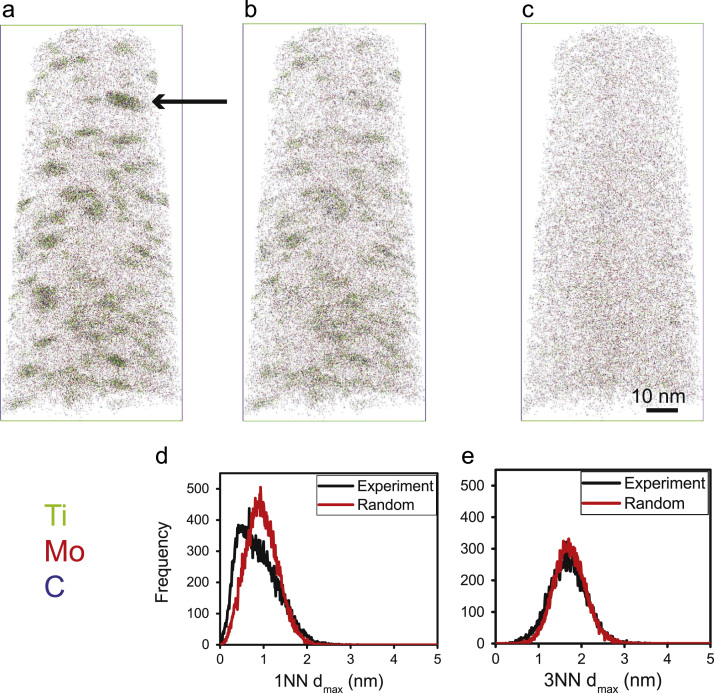
Distribution of solute atoms in the (a) original dataset; and the resulting ‘matrix’ after removing precipitates identified by cluster-finding analyses using a *d*_max_ determined from either a (b) 1NN or (c) 3NN distance frequency distribution of the whole dataset. (d-e) Corresponding NN distribution plots of the remaining matrices.

Fig. 3a shows the comparison between the experimental and random KNN distributions (*K* = 1 and 3) and corresponding cumulative frequency histograms, for the solute elements Ti, Mo and C, all considered together. Higher order distributions (*K* > 3) are not shown as they were found to be similar to the 3NN distribution. Clearly defined bimodal nature is observed in the experimental 3NN plot, which indicates two NN distributions defined by different average NN distance values, belonging to the precipitates and matrix as shown in Fig. 4a. In the present work, the *d*_max_ parameter has been determined at the point where the difference between the experimental and random cumulative plots is greatest, an approach similar to Marceau et al. [7]. The value of K was selected by comparing the KNN distributions of the experimental and random data after removing the clustered atoms defined by *N*_min_ = 30 (i.e. the precipitates). Discussion of choice of value for *N*_min_ will follow soon. Fig. 4 shows a comparison of the 3D atom maps of the original data (Fig. 4a) and that of the remaining data after removing the precipitates by cluster-finding analysis based on *K* = 1 (Fig. 4b) and *K* = 3 (Fig. 4c), using *N*_min_ = 30 in both cases. The corresponding KNN distribution plots from the remaining matrices (precipitates ‘removed’) are shown in Fig. 4d, e. Clearly, cluster-finding analysis for precipitate identification using the 1NN distribution as a heuristic tool, did not effectively account for all the atoms that define the precipitates, since they have not been removed from view (Fig. 4b compared to c) and the remaining ‘matrix’ data still contains a bimodal distribution (Fig. 4d compared to e). Therefore, *K* = 1 is not suitable for the present precipitate analysis. Higher order distributions (*K* = 5 and 7) resulted in similar findings as *K* = 3, and are therefore not included here.

**Fig. 5:**
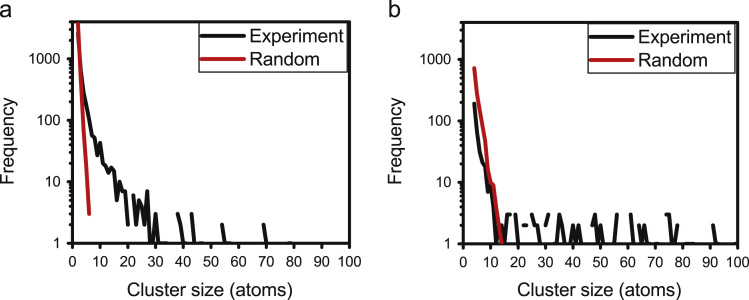
Estimation of *N*_min_ from cluster size distribution analysis. Comparison between experimental and random plots obtained as a result of d_max_ determined from (a) 1NN and (b) 3NN distance frequency distributions. *N*_min_ is assigned to be a value greater than the random cluster size.

The *N*_min_ parameter has been determined from comparison of the experimental and random cluster size distribution plots, available from the IVAS cluster analysis tool [4], where this value is chosen to be greater than the maximum cluster size given by the random plot. It is noted that the value of *N*_min_ determined from this comparison, depends on the order (*K*) of the nearest neighbour distribution as shown in Fig. 5, which compares that from 1NN (Fig. 5a) and 3NN (Fig. 5b). Using this methodology, *N*_min_ has been determined to vary from 20 to 40 when *K* is considered from 3 to 7.

In summary, the choice of *K* and *N*_min_ depends on the preference of the size (number of atoms) of the microstructural features to be analysed in the dataset. The value of the *d*_max_ parameter is critical compared to the other parameters, and so the utmost importance is given to the optimisation of *d*_max_ for each dataset while keeping *K* and *N*_min_ constant for all datasets to be analysed. In the current work, *K* = 3 and *N*_min_ = 30.

### 2.3 Identification of matrix atoms within precipitates

The chemical composition of the precipitates identified by the above method is controlled by the solute atoms selected during determination of *d*_max_. However, the possibility of matrix atoms being situated inside the precipitates cannot be ignored. In the present work, the matrix atoms in the precipitates are identified by introducing additional parameters in the cluster-finding process, known as the double maximum separation method [5], [8], [9]. In this two-stage process, additional atoms within a distance, *L*, of the clustered solute atoms, are included in the clustered entity (in this case, precipitates). This selection of atoms is then eroded back a distance of E (erosion parameter). Values of *L* and *E* are recommended to be close to the *d*_max_ value [5], [10].

**Fig. 6:**
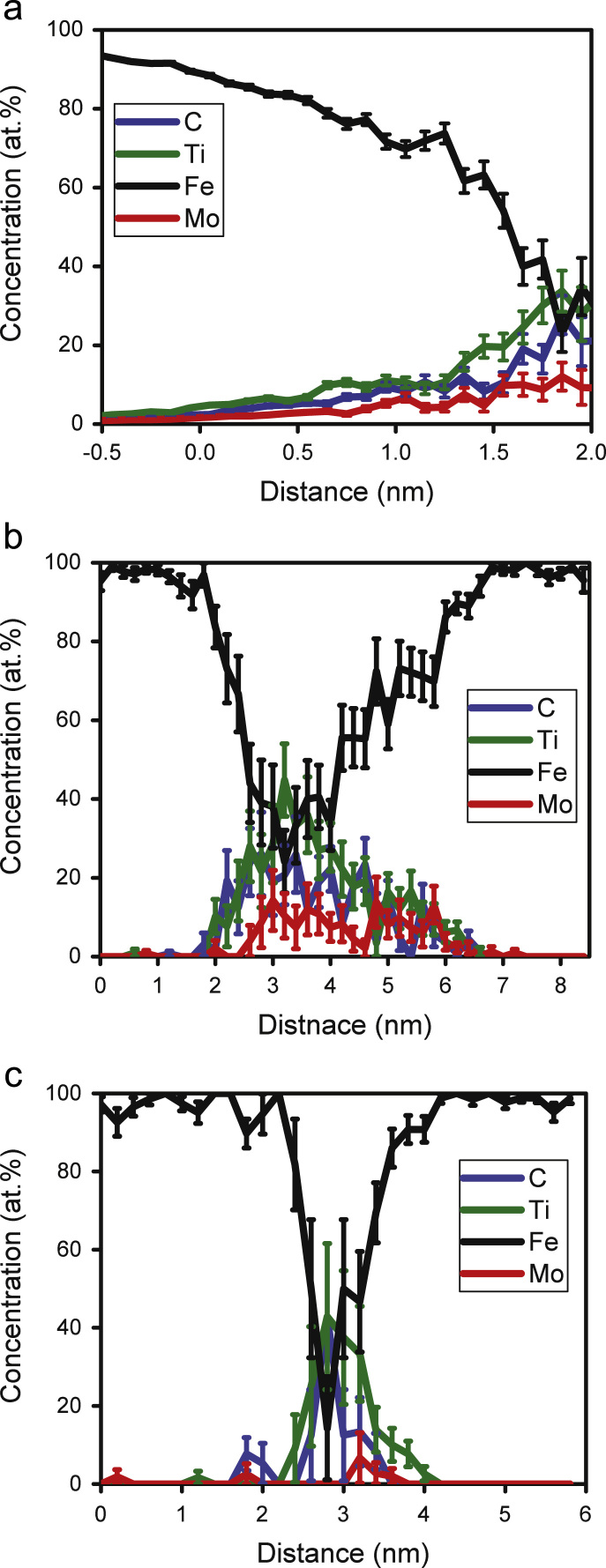
(a) Proximity histogram of all identified particles. One-dimensional (1D) composition profiles of (b) the large particle identified by the arrow in Fig. 4a and (c) a comparatively smaller particle identified in the same dataset. 1D profiles are taken normal to the particle interface and close to the analysis direction (specimen length axis).

In order to study the chemical composition of the precipitate particles, a proximity histogram [11] has firstly been determined for all the particles, employing a 2 at% C isoconcentration surface, Fig. 6a. The Fe content is about 40 at% at a distance from the interface (into the particle) greater than 1.5 nm (radius). The chemical composition and equivalent spherical radius of the precipitate marked by an arrow in Fig. 4a are presented in Table 3 after identification by double maximum separation method and varying the *L* and *E* parameters. The *d*_max_ (0.9 nm) and *N*_min_ (30 atoms) values have been kept constant. The results determined by a ‘bulk composition’ measurement of the same precipitate isolated by a 3 at% Ti isoconcentration surface have also been shown for comparison purpose. Although the results in Table 3 are dissimilar due to inherent differences amongst the analysis approaches, a similarity in the metallic-to-non-metallic atom ratio (Ti+Mo/C) is evident in all three cases. The Ti+Mo/C ratio in the two double maximum separation approaches is approximately 1.8, while in the third case (isosurface method) it is found to be 1.7. Composition determined using proximity histogram analysis is not listed here.

**Table 3:** Chemical composition and equivalent spherical radius of the precipitate identified by an arrow in Fig. 4a, measured by different methods.

**Analysis method**	**Radius (nm)**	**C**	**Ti**	**Fe**	**Mn**	**Mo**
Double maximum separation (*L* = *E* = 0)	1.99	34.9 ± 1.6	44.1 ± 1.7	0	0	20.5 ± 1.3
Double maximum separation (*L* = *E* = 0.9)	2.10	6.5 ± 0.8	8.2 ± 0.9	78.8 ± 1.4	1.9 ± 0.4	3.8 ± 0.6
Isosurface (3 at% Ti)	3.65	13.9 ± 4.1	17.7 ± 4.1	59.7 ± 4.9	2.9 ± 1.5	5.4 ± 2.2

These particles have been previously reported to be (Ti, Mo)-C precipitates [12]. Also, both Ti and Mo have a superior carbide-forming ability compared to Fe [13] so it is therefore less likely for Fe to be actually present in the carbides as the largest metal-atom contributor. One of the possible reasons for detecting such a large amount of Fe inside the precipitates could be due to ion trajectory aberrations caused by differences in the evaporation fields between the Fe-based matrix and the precipitate phase, leading to a local magnification effect [14]. The local magnification effect is not accounted for in the standard reconstruction protocol, leading to artefacts such as displaced atoms [14]. As the effect of local magnification is considered lower along the analysis direction (i.e. in-depth, along the specimen needle axis), the composition of one large and one small particle has been determined along that direction using a one-dimensional (1D) composition profile. The larger precipitate (Fig. 6b) exhibits a substantial rise in solute content (mainly Ti and C) at the core of the precipitate, along with a decrease in Fe content. The 1D composition profile of the smaller particle (Fig. 6c) also shows a drop in the Fe concentration at the core of the precipitate, and despite the fluctuation in these results due to binning of fewer atoms, there is a discrepancy in Fe content on either side of the precipitate, which indicates preferential retention from the high-field precipitate [15].

**Fig. 7:**
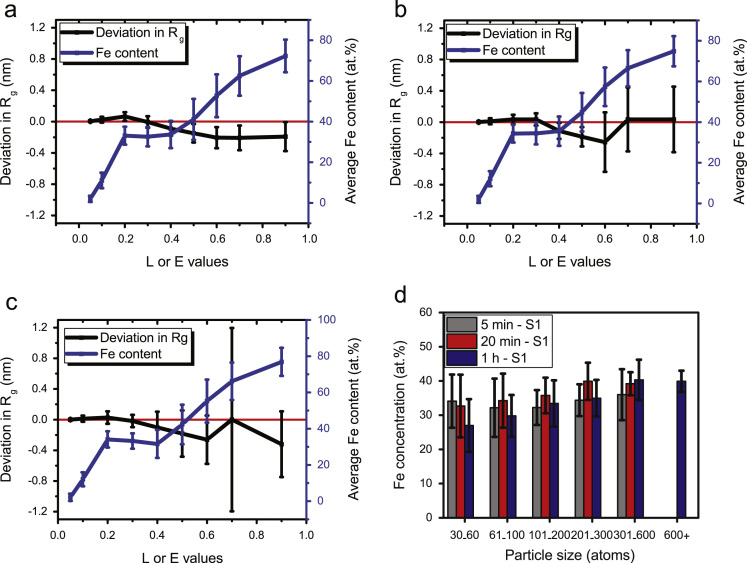
Variation in Fe content and average Guinier radius as a function of *L* = *E* parameters: (a) 5 min – S1 (b) 20 min – S1 and (c) 1 h – S1. Refer to [1] for specimen labels relating to thermomechanical processing conditions. (d) Average Fe content as a function of particle size, obtained with *L* = *E* = 0.3 (error bars represent standard deviation).

Although a significant amount of Fe atoms measured inside the precipitates is likely due to the abovementioned artefact, the presence of Fe inside the precipitates cannot be completely ruled out. Edmondson *et al.* observed a similarly large amount of matrix Fe atoms in Ni-Mn-Si precipitates in an irradiated pressure vessel steel, identified in APT data using the maximum separation method [16]. They compared the compositional profiles obtained by APT with that from scanning transmission electron microscopy paired with energy dispersive spectroscopy (STEM-EDS) and also with STEM-EDS modelling, to confirm about 6 at% Fe present in the precipitates [16]. In the absence of correlative results from other experimental techniques, a different approach was taken in the present work. Firstly, to confirm that the precipitates were influenced by the local magnification effect, variation in the precipitate Fe content as a function of the *L* (= *E*) parameter has been plotted in Fig. 7 for various thermomechanical processing conditions [1]. The deviation in the Guiner radius (*R*_g_) has also been compared (Fig. 7d). The error bars represent the standard deviation of the data. The term “Deviation in *R*_g_” is defined as:

$$∆R_{g}=\bar{R_{g}^{L}}-\bar{R_{g}^{L=0}}$$

Here, $\bar{R_{g}^{L}}$ is the average Guinier radius at different values of *L* = *E* and $\bar{R_{g}^{L=0}}$ is the average Guinier radius when *L* = *E* = 0.

It is evident from the plots in Fig. 7a-c that the variation in Fe content with *L* (= *E*) is similar in all the three conditions. Also, it seems to reach a constant value of around 30–35 at% Fe between *L* (= *E*) values of 0.2 to 0.4 nm. The corresponding $∆R_{g}$ in this range is close to zero with smaller standard deviations. However, the average amount of Fe produced by this method seems rather high compared to the EDS results reported by Wang et al. [17] using a carbon replica TEM sample. On the other hand, Danoix et al. [18] studied precipitation in a Nb-containing model steel using APT and reported a gradual decrease in Fe content of the particles (from 30 at% to less than 10 at%) with increasing aging time. In the current work however, it seems that the average Fe content inside the precipitates is almost constant irrespective of coiling time or particle size (Fig. 7d).

**Fig. 8:**
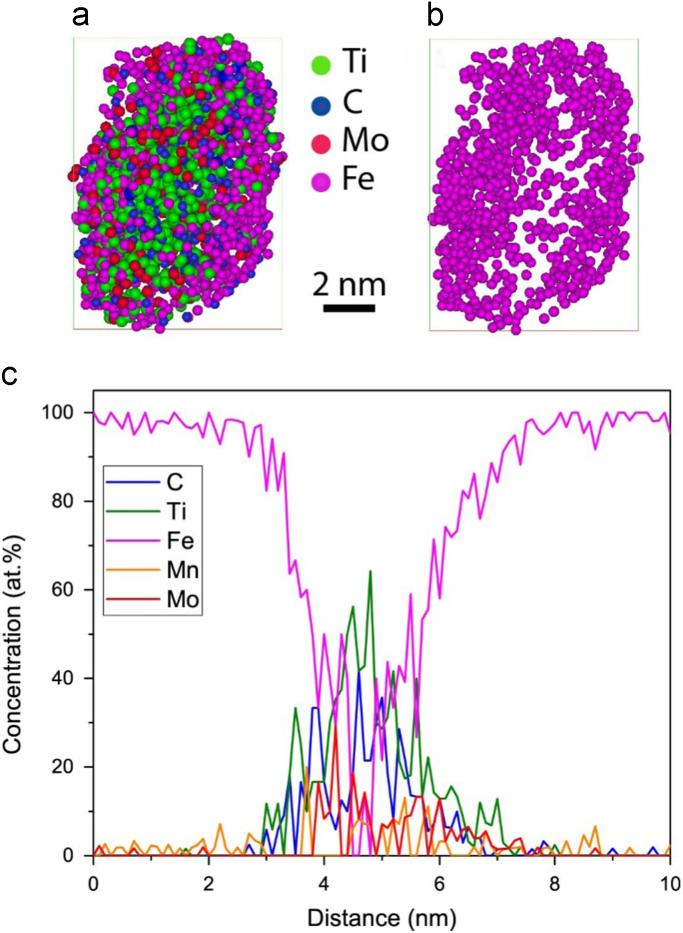
(a) Distribution of atoms in the carbide precipitate (marked by an arrow in Fig. 4), identified by a 1.8 at% C isoconcentration surface; (b) only Fe atoms displayed (note the low-density region around the central area). (c) 1D compositional profile of the precipitate along the analysis (z) direction.

The distribution of Fe atoms in one of the precipitates is shown in Fig. 8a. Concentrated regions of Fe are clearly observed around the periphery (Fig. 8b). The 1D concentration profile along the analysis shown in Fig. 8c quantifies this observation and shows that the amount of Fe becomes negligible in the central region (~1 nm) of the precipitate. Leitner et al. [19] proposed a method to correct the chemical composition of the precipitates considering the effect of local magnification, but it relies on them being suitably aligned along the z direction. In this work however, atomic density of the precipitates and matrix have been compared to that known theoretically to identify the extent of the local magnification effect. Whilst the theoretical matrix density has been determined assuming pure Fe (BCC structure) with a lattice parameter of 0.285 nm, the carbide precipitates are assumed to have a NaCl crystal structure with a lattice parameter of 0.433 nm, as reported by Funakawa et al. [12]. Experimentally, the atomic density of the precipitates was determined by taking the average from the five largest precipitates identified with a 2 at% C isoconcentration surface. The experimental atomic density of the matrix represents the average from five different locations within the reconstructed data, visibly free from particles. Considering detector efficiency (~42%) of the APT instrument used in this study, the theoretical atomic density of both the precipitates and matrix should be about twice that measured from the experimental atom probe data. Whilst this is true for the matrix (Table 4), the theoretical and experimental density of the precipitates seem to be similar, a result of excess Fe atoms identified in the precipitate due to the local magnification effect. Therefore, these excess atoms need to be removed to obtain an accurate chemical composition of the precipitates.

**Table 4:** Comparison of atomic densities (atoms/nm^3^) of matrix and precipitates.

	**Theoretical**	**APT**
**Matrix**	83.3	43 ± 3
**Precipitate**	49.4	38 ± 6

As matrix atoms in the present work have been identified by the L and E parameters in the cluster-finding analysis, corrections in the chemical composition have been applied by modifying these two parameters. For this purpose, the atomic density of the precipitates has been measured after identification with the double maximum separation method using *L* (= *E*) values of 0.1, 0.3 and 0.6 nm, and then compared with the theoretical value. As a result of this process however, the measured density values are inconsistent depending on the *L* or *E* parameters. Therefore, a new parameter, δ, has also been introduced, which is ratio of the density difference between matrix ($D_{\mathrm{Fe}})$and precipitate ($D_{\mathrm{ppt}})$, and the matrix density,

$$\delta =\frac{D_{\mathrm{Fe}}\;-\;D_{\mathrm{ppt}}}{D_{\mathrm{Fe}}}$$

which is expected to be much more consistent.

The comparison of densities at different *L* (= *E*) values with the theoretical precipitate density is given in Table 5, along with the corresponding *δ* values. Reported errors represent standard deviations of the averages. Clearly the average density values as well as the standard deviation increase with *L* (= *E*). On the other hand, the *δ* parameter decreases with increasing *L* (= *E*). The average precipitate density calculated at *L* = *E*= 0.1 nm is closest to the theoretical value taking detector efficiency into account (Table 5) and therefore seems to be the optimum value for these parameters.

**Table 5:** Comparison of average density (atoms/nm^3^) and δ parameter at various *L* (= *E*) values.

**Condition**	**Average density**	**δ**
**Theoretical**	49.4	0.41
***L* = *E* = 0.1**	22 ± 8	0.48 ± 0.20
***L* = *E* = 0.3**	31 ± 13	0.27 ± 0.30
***L* = *E* = 0.6**	78 ± 55	−0.87 ± 1.30

**Fig. 9:**
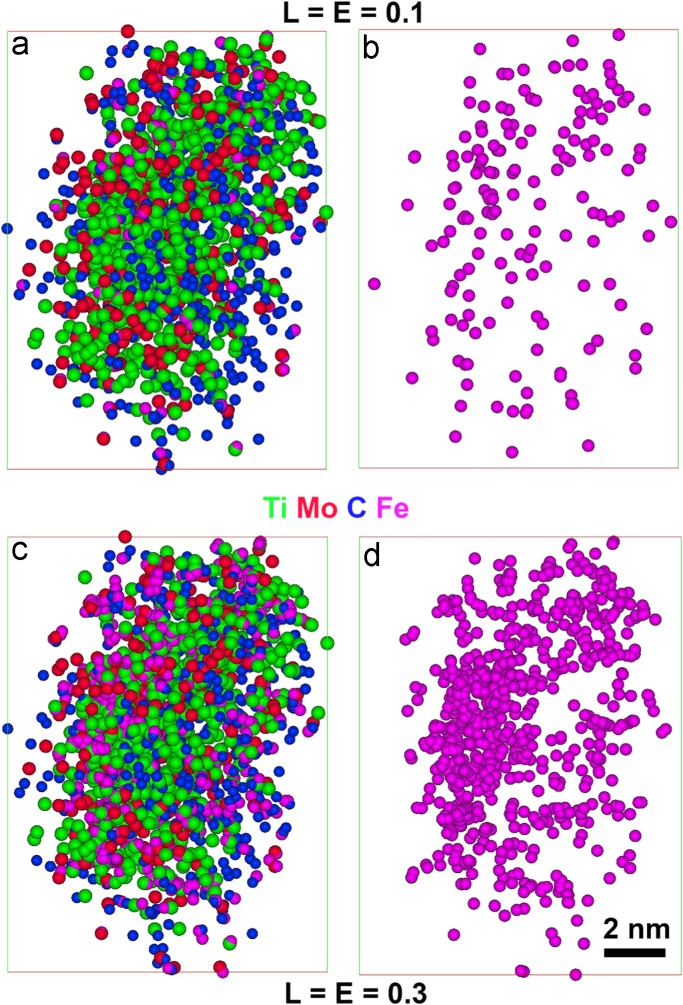
Atomic distribution of the precipitate marked by the arrow in Fig. 4, identified with (a-b) *L* = *E* = 0.1 nm and (c-d) *L* = *E* = 0.3 nm, and viewed along x–y plane, perpendicular to the analysis (z) direction.

The atomic distribution of solute elements and Fe atoms in the precipitate marked by the arrow in Fig. 4 is shown in Fig. 9 when identified by different *L* (= *E*) parameters (0.1 and 0.3 nm). The distribution of Fe atoms at *L* = *E* = 0.3 nm (Fig. 9d) is non-uniform, similar to the observation in Fig. 8b. In contrast, a uniform distribution of Fe has been observed in the precipitate identified with *L* = *E* = 0.1 nm (Fig. 9b). Considering both the average atomic density and Fe atom distribution of the precipitates, this latter value has been applied in all cases for precipitate-finding analysis.

### 2.4 Cluster-finding analysis for solute clusters

The possibility of the existence of smaller particles than the precipitates (termed clusters in [1]) has also been investigated, where these clusters were defined to consist of ≤ 30 atoms. It is important to note the possibility of ‘misidentifying’ such small particles as random compositional fluctuations in the solid solution, and this will be discussed later in this section. The cluster-finding approach used in the present work for identification of solute clusters is comparable to that taken to identify the precipitate particles, and employs the previously described maximum separation method (i.e. without the envelope and erosion process). In this case however, the cluster-finding analysis was performed on the remaining matrix after removal of the precipitates from the whole dataset. The order of the NN distribution was chosen as *K* = 1, with an *N*_min_ value of 2 atoms. Again, the *d*_max_ parameter was chosen at the point of maximum difference between the cumulative frequencies of the NN distance distributions of the experimental and randomly labelled data, similar to the process for precipitate particle identification.

To avoid the systematic effect of erroneous detection of solute clusters due to limited detector efficiency [20], and to quantitatively appreciate the extent to which the data contains solute clustering beyond that expected from random compositional fluctuations in the solid solution, experimental cluster-finding results have been compared to that from the corresponding random dataset, following Marceau et al. [7]. This ‘experimental-minus-random’ approach allows robust analysis where other methods such as visual inspection of the 3D atom map or construction of isosurfaces (isodensity or isoconcentration) are not suitable, owing to the small size of the solute cluster particles.
